# Exercise Training Prevents Endometrial Hyperplasia and Biomarkers for Endometrial Cancer in Rat Model of Type 1 Diabetes

**DOI:** 10.4021/jocmr444e

**Published:** 2010-10-11

**Authors:** Muhammed Al-Jarrah, Ismail Matalka, Hasan Al Aseri, Alia Mohtaseb, Irina V Smirnova, Lesya Novikova, Lisa Stehno-Bittel, Ahed AlKhateeb

**Affiliations:** a Department of Allied Medical Sciences, Faculty of Applied Medical Sciences, Jordan University of Science and Technology, Jordan; b Department of Pathology, Faculty of Medicine, Jordan University of Science and Technology, Jordan; c Department of Toxicology and Forensic Sciences, Jordan University of Science and Technology, Jordan; d Department of Physical Therapy and Rehabilitation Science, University of Kansas Medical Center, USA

## Abstract

**Background:**

Endometrial cancer is one of the most common types of gynecologic cancers. The ability of exercise to reduce the risk of endometrial cancer in women with type 2 diabetes has been established, but no studies have examined this link in type 1 diabetes.A randomized, controlled animal study was designed using a standard rat model of type 1 diabetes. The goal of this study was to investigate the ability of exercise to prevent increased levels of endometrial cancer biomarkers, estrogen receptor (ERα) and p16, and endometrial hyperplasia associated with diabetes.

**Methods:**

Forty female rats were randomized into four groups: sedentary control, exercise control, sedentary or exercised diabetic. Diabetes was induced by alloxan injection. A 4-week treadmill training program was initiated with the development of diabetes. Endometrial tissues were evaluated for hyperplasia and ERα and p16 levels and subcellular localization using microscopy.

**Results:**

Severe diabetes lead to hyperplasia in the endometrial tissue in 70% of sedentary diabetic rats. Exercise-trained diabetic rats and the non-diabetic rats displayed no hyperplasia. The expression of ERα increased significantly (p < 0.02) while the expression level of p16 decreased significantly (p < 0.04) in the diabetic sedentary group compared to the non-diabetic groups. Exercise training led to a reversal in the percentage of p16 and ERα positive cells in diabetic rats.

**Conclusions:**

Severe diabetes leads to hyperplasia of the endometrial tissue and increased ERα levels and decreased p16 levels in rats, which can be prevented with aerobic exercise.

**Keywords:**

Diabetes; Estrogen receptor alpha; P16; Endometrial hyperplasia; Endometrial cancer; Exercise

## Introduction

Endometrial cancer is the fourth most common type of cancer among women and the most common gynecologic cancer [1, 2]. Twenty-two percent of woman who have had endometrial cancer will experience recurrences [3]. Most startling, endometrial cancer incidence and mortality rates are on the rise in spite of the fact that most cases are diagnosed at early stages [4]. Although the exact cause of endometrial cancer is unknown, there are many factors that increase the risk of endometrial cancer, including chronic diseases such as diabetes [5, 6]. Most studies have focused on the link between type 2 diabetes and endometrial cancer, finding that the risk of endometrial cancer rises with greater levels of obesity [7, 8]. However, this association has not always been clear as one study reported that a body mass index (BMI) of greater than 35 was not associated with endometrial cancer, while a lower BMI was associated [6]. A prospective study analyzing 24,000 post-menopausal women found that diabetes, independent of obesity, was associated with an increase in the risk of endometrial cancer [8]. It has been hypothesized that changes in insulin levels may be part of the link between diabetes and cancer [9].

While the link between obesity, type 2 diabetes and endometrial cancer has been studied extensively, few studies have focused on the link between the autoimmune form of diabetes, type 1 diabetes, and endometrial cancer [10]. Those that have looked at the risk and prognosis of endometrial cancer specifically in women with type 1 diabetes have determined that there is an association [10-12]. Zendehel et al. studied the increased risk for all cancer in people with type 1 diabetes. They found that people with type 1 diabetes had elevated risks of cancers of the stomach, cervix and endometrium [13]. Clearly, there is a need for more controlled studies that specifically examine the mechanistic link between diabetes and endometrial cancer [14].

In 2001, the International Agency for Research on Cancer published a report concluding that physical activity could reduce the risk of endometrial cancer [15]. Some of the most important data suggests that sedentary behavior is a risk factor for endometrial cancer that is independent of obesity [16]. Such studies also suggest that women who are physically active have a 20 percent reduced risk of endometrial cancer [17]. The current study is the first to focus on the molecular changes that occur in the endometrium using an animal model of type 1 diabetes. The purpose of this study was to determine whether there was a change in biomarkers for endometrial cancer in an animal model of type 1 diabetes. The effects of exercise, an intervention known to significantly lower the risk of endometrial cancer in women [17-19], was tested to determine whether it could prevent early endometrial changes in animals with type 1 diabetes.

## Materials and Methods

### Animals

Eight-week old Sprague-Dawley female rats were used in this study and randomly assigned into one of four groups. Sedentary control (SC, n = 10), exercise control (EC, n = 10), sedentary diabetic (SD, n = 10), exercise diabetic (ED, n = 10). Animals were housed in individual cages at a 22 + 1^o^C in a controlled room with a 12:12 light: dark cycle. The animals were allowed free access to standard chow and water. All experiments were performed in accordance with the institutional research committee guidelines for animal experimentation.

Rats in the two diabetic groups received an intraperitoneal injection of alloxan (120 mg/kg) or saline in the control groups. Three days later, a confirmation of successful induction of diabetes was assessed by demonstration of hyperglycemia in the rats. Animals with fasting blood glucose above 250 mg/dl were classified as diabetic animals.

Animal Care and Use Committee Approval obtained from the Jordanian University of Science and Technology.

### Exercise protocol

The exercise training protocol utilized was based on previous publications providing evidence of adequate systemic and cellular adaptations with this level of aerobic exercise [20, 21]. Aerobic exercise training was conducted on a custom treadmill with 8 separate lanes. Following the alloxan or saline treatment, the rats in the exercised groups were introduced to the treadmill slowly over the course of a week with initial orientation and walking on the moving treadmill. The 4 week exercise protocol did not begin until rats could run at a speed of 18 m/min. The training protocol was individualized for each animal, but in general consisted of running for 40 min/day for 5 days/week at a speed of 18 m/min. Sedentary rats did not exercise; however, they were transported daily to the training room so that they were exposed to the same environment as the exercised groups of animals.

### Immunostaining of p16 and estrogen receptor (ERα) in the endometrium

Tissues were collected from the stromal and epithelial regions and fixed in 4% parafolmaldehyde and subsequently embedded in paraffin. Sections (3 - 4 μm) were mounted on coated slides (microslides sc-24976, Santa Cruz). Prior to staining, samples were deparaffinized in xylene twice for 2 minutes, then rehydrated through serial dilutions of alcohol (100%, 90%, 80%, and 70%) ending in water (2 minutes each step). This was followed by treatment under pressure in the reveal solution (RV1000M, Biocare Medical, Concord, CA) for 2 minutes in the Decloaking chamber (Biocare Medical) in order to retrieve the antigens, and later to block endogenous biotin. After cooling to room temperature, sections were incubated with 3% hydrogen peroxide in methanol in order to block the endogenous peroxidase activity for 5 minutes and then were washed in phosphate buffered saline (PBS). According to the manufacturer's instructions, sections were incubated for 1 hour at room temperature with ERα (Santa Cruz Biotechnology, Santa Cruz, CA) and p16 (Biocare Medical, San Antonio, TX) antibodies which were diluted according to vendor instructions. Subsequently sections were washed in PBS and incubated with biotinylated secondary antibody (LSAB kit, Dako Carpinteria, CA) for 15 minutes at room temperature, then washed with PBS. Sections were incubated with streptavidin horse radish peroxidase (LSAB kit, Dako) for 15 minutes at room temperature and washed with PBS. 3,3'-Diaminobenzidine (DAB) substrate was applied for 2 minutes or longer, until the desired color intensity was developed, and then the slides were washed with tap water to stop the reaction. Throughout the study, sections from normal human endometrium known to express the investigated proteins were analyzed in parallel to serve as positive controls. Omission of the primary antibody from these samples served as negative controls. All sections were counterstained with hematoxylin and examined by light microscopy. Ten slides from each animal group were evaluated for p16 and ERα expression by two blinded independent evaluators. The area labeled with specific antibody and the percentage of cells positive for the antigen were calculated using published procedures [22]. The evaluation of immunostaining of ERα and p16 was performed on both the stromal and epithelial components, but only statistical analysis of the stromal regions were reported. Findings in the epithelial regions presented with wide variability that failed to show statistically significant differences.

### Examination of tissue pathology

Ten slides were randomly assigned for histopathology evaluation from each group of animals. Haematoxylene and Eosin staining was done using standard protocol. Two double blinded pathologists evaluated the sections for estrous cycle stage and indications of hyperplasia from the four experimental groups. The hyperplasia was evaluated and graded using standard procedures according to the number, crowding and complexity of glands, the glands to stroma ratio, and epithelial atypia [23].

### Data analysis

One way ANOVA was completed on all animals followed by paired and unpaired student t-test analysis to determine statistical significance within each group and to compare two groups. Kruskal-Wallis One Way ANOVA on Ranks was completed for histological analysis of glandular formation followed by a Dunn’s Test. Differences at p value < 0.05 were considered statistically significant.

## Results

### Animal characteristics

As expected, the 8-week old rats continued to grow after the induction of diabetes. There was no statistically significant difference in the percentage of gained weight for any group, which ranged from 7 to 17% increase from their initial body weight (Table 1). At the completion of the study, rats from the two diabetic groups had significantly higher blood glucose levels than the two control groups (p < 0.001, Table 1). Interestingly, the diabetic rats that ran on the treadmill had a significantly lower blood glucose level at the termination of the study than the sedentary diabetic rats (p < 0.001, Table 1).

**Table 1:** Animal Characteristics

Group	Initial Weight (g)	Final Weight (g)	Blood glucose (mg/dl)
Sedentary Control (SC)	120 ± 11	143 ± 8	104 ± 6
Exercise Control (EC)	131 ± 12	158 ± 13	94 ± 9
Sedentary Diabetic (SD)	125 ± 10	134 ± 7	430 ± 13^*^
Exercise Diabetic (ED)	128 ± 11	152 ± 12	237 ± 10^*#^

### Expression of ERα in the endometrium

**Figure 1.**
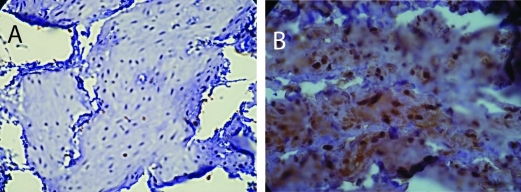
Immunohistochemistry for ERα. A) Section from a sedentary control rat showing positive staining for ERα predominantly in the nuclei. B) Section from sedentary diabetic rat illustrates a considerable increase in the ERα positive cells with both a nuclear and cytoplasmic distribution of the protein with more intense levels. Counterstaining was performed with hematoxylin.

**Figure 2.**
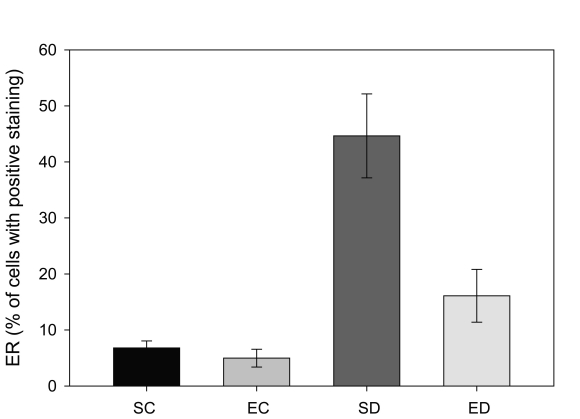
Comparison of estrogen receptor (ERα positive staining in four groups of rats. Tissue from sedentary control (SC) and exercised control (EC) rats illustrated less than 10% of the total area that was positive for ERα per region analyzed. Sedentary diabetic (SD) animals showed a marked increase in ERα immunostaining (* indicates difference from the three other groups, p < 0.02), with exercise training (ED) showing a trend towards reversal.

Estrogen receptor (ERα) levels have been shown to be highly correlated with gynecological cancers, and have been suggested as simple screening mechanism for high risk patients [24]. In the rats with diabetes from this study, there was a significant increase in the expression of ERα in the endometrium. Figure 1 shows two representative fields from control sedentary and diabetic sedentary animals. ERα levels were analyzed in stromal cells during the mid-secretory phase of the cycle when baseline ERα levels are at their lowest [25]. Control tissue had limited ERα staining in the nuclei of stromal cells, while sections from diabetic animals showed staining in both the cytoplasmic and nuclear regions of the cells, along with other indications of proliferation. Since both the amount of immune-reactive tissue and the number of ERα-positive cells are important indicators of protein levels, two quantization methods were utilized. First, the total area of ERα staining within each micrograph was calculated as a percentage. Figure 2 demonstrates a robust increase in the total ERα-stained area in the sedentary diabetic (SD) group of animals, which was statistically different from the two control groups (p < 0.02). There was greater variation in the area of ERα staining in the samples from the sedentary diabetic group with some stromal regions reaching as high as 82%. Treadmill exercise induced no change in the level of ERα in the control groups (comparing sedentary and exercised controls). The exercise protocol did reduce the area stained positive for ERα in the diabetic group, but not to a statistically significant level.

The same trends were noted when micrographs were analyzed for the percentage of cells positive for ERα, while this procedure excludes analysis of increased protein levels within cells. There was no difference in the percentage of cells positive for detectible ERα levels between the sedentary and exercised control groups. However, there was a 12% increase in the ERα-positive cells in samples from the diabetic sedentary compared to the control sedentary group (p < 0.04). Exercise training reduced the percentage of cells that were ERα-positive in the stromal region of diabetic animals, but not to statistically significant levels.

### Expression of p16 in the endometrium

**Figure 3.**
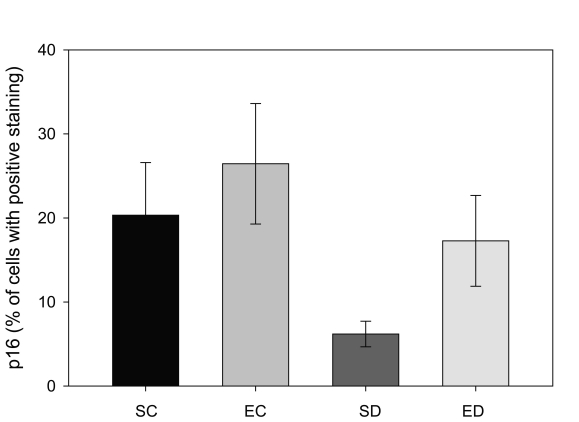
Comparison of p16 positive staining in four groups of rats. Tissue from sedentary control (SC) and exercised control (EC) rats had a greater area of p16-positive immunostaining. Diabetes (SD) reduced the amount of p16-staining when compared to the other three groups (* indicates p < 0.04), while exercise training of the diabetic animals (ED) increased the amount of p16 so that it was not statistically different from the control groups (# indicates significant difference between SD and ED).

The p16 protein, a CDKN2A gene product, is known as a tumor suppressor protein. Decreases in the level of p16 have been linked with cancer, especially uterine and endometrium [26, 27]. Cells with positive staining for p16 from the control rats were found to have predominantly cytoplasmic staining with little nuclear localization, as described previously [28]. In contrast to the increase in the ERα levels in diabetic rats in this study, the amount of p16 decreased significantly from 20% of the total tissue area in the sedentary control group to 6% in the sedentary diabetic group (Figure 3, p < 0.04). The results showed that treadmill exercise training increased the area staining positive for p16 in the non-diabetic group from 20% to 26%, which was not a significant change (p < 0.53). In contrast, treadmill exercise training had a significant impact on the p16 levels of the endometrium from diabetic rats. The training protocol resulted in an increase in the area of positive p16 immunoreactivity from 6% to 17%, which was statistically significant (p < 0.04).

### Histopathology results

**Figure 4.**
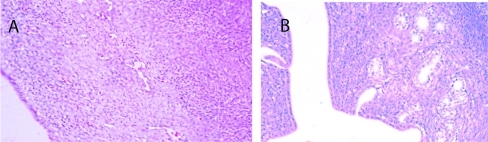
Histological analysis of endometrial tissue. A) Typical section from a sedentary control rat. B) Typical section from a sedentary diabetic rat showing excessive glands (arrows) with tubular cells surrounding the glands, along with signs of hyperplasia.

**Figure 5.**
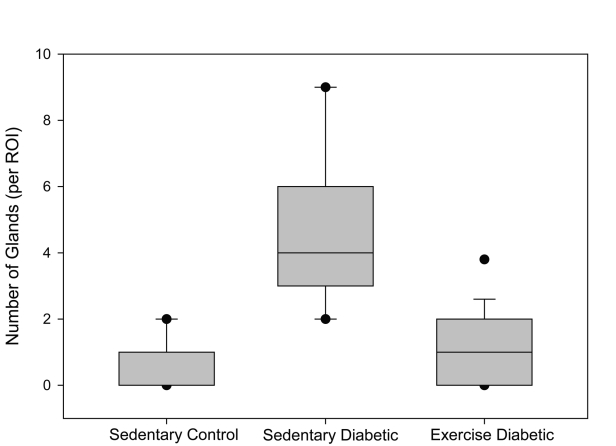
Number of glands. The box plot depicts the median (bold line within the box) number of glands per region of interest (ROI) within the endometrial stroma and the 25th and 75th percentiles (box) between sedentary control, sedentary diabetic and exercised diabetic animals. Vertical lines (whiskers) indicate the 5th and 95th percentiles with outliers shown by the circles.

Seventy percent of the specimens analyzed from the sedentary diabetic group showed elements of simple glandular hyperplasia including excessive endometrial glands. No hyperplasia was detected in the control groups or the exercised diabetic group. Figure 4 provides examples of stained specimen from the two diabetic groups (sedentary and exercised). The images show excessive glands and tubular cells with large nuclei typical of simple hyperplasia in sections from the sedentary diabetic animals. Endometrium from the non-diabetic groups was comprised of healthy spindle-shaped cells. The sedentary control and exercise-trained diabetic groups had means of 0.52 ± 0.14 and 0.98 ± 0.26 glands per region of interest, respectively (Figure 5). Tissues obtained from the sedentary diabetic group had an average of 5.21 ± 0.75 glands, signifying nearly ten times more glands per area when compared to the other groups (p < 0.001). None of the sections examined contained tumors.

## Discussion

Most work linking diabetes to cancer risk has focused on type 2 diabetes and obesity. The risk clarified in meta-analysis shows a clear increase in mortality from all causes in cancer patients who had preexisting diabetes [29]. Specifically the prognosis for women with endometrial cancer and diabetes is worse than those without diabetes as it correlates to a higher rate of lymph node spread and poor overall survival [30]. When analyzed for the types of cancer that were a risk factor in people with diabetes (type 1 and type 2), endometrial, breast and colorectal cancer were associated with the highest risk [29].

The purpose of this study was to test the link between endometrial changes indicative of cancer in a type 1 animal model of diabetes, and to determine whether exercise could influence the outcome. In this model, there were clearly hyperplastic changes in the endometrium of the diabetic animals. Specifically, the tissue showed excessive glands surrounded by tubular cells with large nuclei, many of them positive for excessive ERα.

Estrogen plays a central role in regulating growth kinetics of a variety of epithelial linings, most importantly in the endometrium. Estrogen binds to the estrogen receptor (ERα), directly stimulating proliferation and differentiation. ERα translocates to the nucleus, where it binds to promoter sites and thus, regulates the expression of many genes [31]. At a cellular level, estrogen has an immense impact on which genes are turned on or off, leading to differences in protein levels. This is especially true to type I classifications of endometrial carcinomas, which occur most commonly in premenopausal and perimenopausal women and are estrogen sensitive [32]. Results described here quantify an increase in ERα levels in cells within the endometrium of diabetic rats with both the nuclear and cytoplasmic regions staining positive for the protein.

The p16 has been shown to be an important biomarker for cancerous cells in several types of tumors [33]. In fact, the location of p16 in the cytoplasm versus nuclear compartments can differentiate between different types of endometrial carcinomas [33]. Loss of p16 has been associated with type II endometrial cancer in women [34], and an increased rate of metastases [35] and death [36]. Thus, increased levels of p16 in the diabetic animals that were exercise-trained and the according absence of endometrial hyperplasia in the same group of animals is a significant finding of this study, although it does not indicate a cause/effect relationship.

The steps linking the pathology of type 1 diabetes to the hyperplastic changes noted in the endometrium of sedentary diabetic animals is unknown. Mitochondrial dysfunction is one cellular alteration common to diabetes and most cancers [37]. It is known that there is extensive cross-talk between the fuel metabolism pathways and apoptotic molecules. Moreover, unbalanced organelle stress associated with ER stress is present in both diabetes and cancer [38, 39]. Since type 1 diabetes is an autoimmune disease, altered immune function offers another link between the disease and cancer with several different immune cells proposed as the culprit [40, 41].

An obvious link between diabetes and hyperplasia is in the increased glucose availability that is a hallmark of cancer cells, and part of the so-called Warburg effect [42-44]. It is possible that high glucose levels associated with diabetes could have stimulated the hyperplasia noted in the tissue analyzed in this study, although most researchers agree that the link between glucose and cellular transformation is more complex. Following this line of reasoning, one possible cause for the prevention of the hyperplastic changes in the specimens with exercise training may be due to the decreased mean blood glucose levels in the trained animals. Table 1 shows a statistically significant 45% decline in the blood glucose levels at the end of the four week exercise training protocol. Such a decline theoretically could prohibit the proliferative phenotype of the cells.

In conclusion, the results from the current study showed that 1) endometrial estrogen receptors, which can be carcinogenic, were present in higher levels with diabetes and that exercise training showed a trend to reverse it; 2) levels of p16, a known tumor suppressor, were decreased with diabetes, but reversed when the diabetic animals exercised; 3) endometrial hyperplasia was noted in 70% of the samples from the sedentary diabetic rats, but not in the exercise trained animals. It is important to note that, although the risk for endometrial cancer is greater in patients with type 1 diabetes, the total risk is still low when compared to other more common complications of type 1 diabetes. While this study found hyperplastic changes in the endometrium of rats with uncontrolled type 1 diabetes, no solid tumors were identified. The mechanisms linking the systemic influences of type 1 diabetes with endometrial hyperplasic and possibly endometrial cancer, or any other cancer, are not fully elucidated, but likely include both p16 and the ERα. This area of study certainly deserves more attention. The alloxan-injected diabetic rat tested in this project provides a suitable animal model for further mechanistic studies.
